# Distribution of the Soil PAHs and Health Risk Influenced by Coal Usage Processes in Taiyuan City, Northern China

**DOI:** 10.3390/ijerph17176319

**Published:** 2020-08-31

**Authors:** Rongjie Li, Mingchao Cheng, Yang Cui, Qiusheng He, Xiaofang Guo, Laiguo Chen, Xinming Wang

**Affiliations:** 1 School of Environment and Safety, Taiyuan University of Science and Technology, Taiyuan 030024, China; tyustlrj@163.com (R.L.); cheng.mingchao@foxmail.com (M.C.); cuiyang@mail.iap.ac.cn (Y.C.); guoxiaofang@tyust.edu.cn (X.G.); 2 Center of Urban Air Pollution, South China Institute of Environmental Science (SCIES), Ministry of Ecology and Environment of the People’s Republic of China, Guangzhou 510655, China; chenlaiguo@scies.org; 3 State Key Laboratory of Organic Geochemistry, Guangzhou Institute of Geochemistry, Chinese Academy of Sciences, Guangzhou 510640, China; wangxm@gig.ac.cn

**Keywords:** PAHs, spatial distribution, positive matrix factorization (PMF), coal-related sources, carcinogenic risk

## Abstract

The quality of urban soil is closely related to the safety of public places and the guarantee of food quality. This study investigated the level, distribution, source, and carcinogenic risk of 16 U.S. EPA (Environmental Protection Agency) priority polycyclic aromatic hydrocarbons (PAHs) in urban, agricultural, and montane soil in Taiyuan. The ∑16PAHs level varied from 104.78 to 6594.63 ng g^−1^ with a mean of 922.93 ng g^−1^, and 47.73% of the soil samples were severely contaminated, with a concentration higher than 600 ng g^−1^. PAHs with higher molecular weight (≥4 rings) were dominant in PAHs profiles accounting for 80.92%. In the spatial distribution of PAHs, hotspots of ∑16 PAHs were observed near the industries, indicating pollutants emitted by the industries directly affect the surrounding soil quality. The sources identified by positive matrix factorization (PMF) indicated: coal combustion (40.77%), vehicle exhausts (32.94%), biomass combustion (14.89%), and coking source (11.40%). Coal-related sources (coal and coking sources) were the major contributors (52.17%) to PAHs and carcinogenic risk (46.48%) assessed by BaP toxic equivalent concentration in total soils. Therefore, the extensive usage of coal was the leading factor for PAH pollution and health risk in Taiyuan soil.

## 1. Introduction

Over the past decades, with the acceleration of urbanization and the vigorous development of economy in China, a large number of pollutants emitted by energy consumption and human activities lead to increasingly serious environmental pollution. Soil pollution has become an important problem to be solved urgently in China. According to the bulletin of the National Soil Survey, 16.1% of soil samples were polluted by pollutants such as heavy metals and polycyclic aromatic hydrocarbons (PAHs) in different degrees [1]. Poor mobility and degradation result in long-term retention of pollutants in soil. Accidental ingestion, dermal contact, and inhalation were the direct ways for PAHs enriched in soil to enter the human body [2]. At the same time, they could also be indirectly ingested through the food chain [3], resulting in endocrine disorders and even cancer [3,4].

PAHs are a class of the notorious organic pollutants with toxicity, carcinogenicity, and mutagenicity, which threaten human health and have attracted widespread public attention [2,3,5,6,7]. Coal usage was one of the principal sources of PAHs in China [8]. Meanwhile, the heavy usage of coal was also the leading factor resulting in higher PAH levels in urban soils in the north of China (1467 ng g^−1^ in the northeast and 911 ng g^−1^ in the north) than that in the east (737 ng g^−1^), south (349 ng g^−1^), and west (209 ng g^−1^), respectively [9]. Shanxi Province was a typical heavy industry province in northern China, with its coal production ranked first in China and the world. Coal was widely used in industrial activities (such as thermal power plants, chemical works, coking plants, and steel mills) and the daily lives of residents (such as heating and cooking). A large number of pollutants were discharged, including PAHs. It was reported that the amount of PAHs emitted by Shanxi Province in 2003 reached 2430 tons, ranking second in the country [8]. Some studies had reported that the soil in Shanxi Province has been contaminated with extensive and severe PAHs. For example, a total of 16 PAH concentrations (ranging from 250.49 to 9387.26 ng g^−1^, with a mean of 2781.42 ng g^−1^) were observed in agricultural soil near a chemical plant [10], which was three times higher than that of Shanghai (Pudong) (mean value: 807 ng g^−1^) [11]. A study conducted on farmland soils in the coking base reported that the ∑16PAHs concentration of 58.4% samples was higher than 600 ng g^−1^ [12], which was at a serious level of pollution. In addition, there were related reports in City of Changzhi [13], Xiangfen [14], and Xinzhou [15]. These studies were mainly limited to arable soil, and there was a lack of reporting on other types of soil pollution, such as soils in public places that were closely related to public health. Furthermore, the urban and rural effects on mountains were less discussed even if some studies investigated diverse soil [16].

As the capital of Shanxi Province, Taiyuan was one of the cities with the worst air quality in China and even the world [6]. Our previous studies found that high concentrations of PAHs were detected in the atmosphere of Taiyuan [5,6,7]. PAHs mainly entered the soil by dry deposition in Taiyuan, and the PAHs particle dry deposition flux of 6445.95 ng (m^2^ d)^−1^ was much higher than that of Beijing and Tianjin [7]. Soil exposed to such a high level of airborne PAHs could pose a threat to soil and food safety and even public health. However, there were few reports of soil PAHs in Taiyuan. The objectives of this study were (1) to report PAH concentrations in urban, agricultural, and montane soils in Taiyuan; (2) to determine the spatial distribution of PAHs and influential factors; (3) to elucidate the sources of PAHs in the urban area and surrounding areas; (4) to evaluate the health risks of PAHs from identified sources.

## 2. Materials and Methods

### 2.1. Study Region and Soil Sampling

Taiyuan, the capital of Shanxi Province, was a typical coal-based city in north China with the industrial structure of traditional heavy polluting plants, including coal, coking, machinery, power plants, chemical industries, and so on. Moreover, Taiyuan is located in the continental interior with a warm temperate continental monsoon climate. Located in a basin region surrounded by the Lvliang Mountains in the west and the Taihang Mountains in the east, dispersion and dilution of pollutants in Taiyuan were adversely affected. Taiyuan has announced that heavy polluting industries have been relocated to the suburbs under centralized management since 2011, there were still heavy polluting enterprises, including iron and steelwork, heavy machinery plants, and thermal power plants [6]. 

A total of 44 surface soil samples from urban and surrounding areas were collected in April 2016: 15 from urban parks (urban: U1–U15), 19 from agriculture (agriculture: A1–A19), 10 from mountains (mountain: M1–M10). Each site was at least 100 m away from pollution sources and the main roads, without anthropogenic disturbance activities (such as digging, covering, irrigation, and crop straw burning). GPS (Global Position System) was used to record the coordinates of the sampling site location. For each sampling site, 5 sub-samples (each 1 kg) were taken from the same area (at a depth of 0–20 cm in a 100 m^2^ square area) with a stainless steel shovel, and then bulked together to form one composite sample. A quarter of the composite sample was collected and stored in a polyethylene bag. Soil samples were freeze-dried for 48 h, then sieved to 70-mesh-size particles after removing stones and residual roots. All of the samples were kept at −4 °C until analysis. Total organic carbon (TOC) in soil samples was obtained by soil weight difference between before and after ignition at 550 °C in a muffle furnace. 

### 2.2. Chemical Analysis

A 10 g of sample was mixed with 10 g of anhydrous sodium sulfate and 2 g of activated copper sheet in a 150 mL beaker, spiked with 50 μL of standard deuterated PAH mixture as surrogates (20 ppm, d8-Nap, d10-Ace, d10-Phe, d12-Chr, d12-Pyr). The mixture was extracted with ultrasound for 10 min (3 times, stirring before each ultrasound to avoid soil hardening) after 30 mL of n-hexane/acetone (V:V = 1:1) was added. Then, extracts were condensed to 2 mL with a rotary evaporator and loaded onto a silica gel aluminum oxide chromatographic column for cleanup. The chromatographic column was packed from bottom to top with 12 cm silica gel, 6 cm alumina, and 1.5 cm of anhydrous sodium sulfate. The eluent was solvent-changed and concentrated to 1–2 mL before analysis. The final volume was adjusted to 1 mL under a gentle stream of nitrogen, before 1 μL of hexamethyl benzene was spiked as internal standards. Next, 16 target PAHs were detected by Shimadzu GC-MS 2010 plus with RTX-5MS (length 30 m, i.d. 0.32 mm, film thickness 0.25 μm), and helium was used as the carrier gas with a flow rate of 4.24 mL min^−1^. Sample extract (1 μL) was injected in split mode, and the split ratio was set as 3. The oven temperature program was as follows: the initial temperature of 60 °C was held for 4 min, increased at a rate of 6 °C min^−1^ to 300 °C and then held for 30 min. The injector and ion source were maintained at 300 and 200 °C, respectively. Ionization was carried out in the electron impact (EI) mode at 70 eV, and data were acquired using the selective ion monitoring (SIM) mode. PAHs were identified by the selected ions and comparison of the relative retention time between standard solution and samples.

The target PAHs were naphthalene (Nap), acenaphthylene (Acy), acenaphthene (Ace), fluorene (Flu), phenanthrene (Phe), anthracene (Ant), fluoranthene (Fla), pyrene (Pyr), benzo[a]anthracene (BaA), chrysene (Chr), benzo[b] fluoranthene (BbF), benzo[k] fluoranthene (BkF), benzo[a] pyrene (BaP), indeno[1,2,3-cd] pyrene (Ind), dibenzo[a,h] anthracene (DahA), benzo[g,h,i] perylene (BghiP).

### 2.3. Quality Assurance/Quality Control

The instrument was calibrated daily with standard solution and the relative standard deviation of the standard curve was less than 20%. Recoveries of soil samples for 5 deuterated PAHs ranged from 48.14% to 123.88%, including d_8_-Nap (48.14–82.68%), d_10_-Ace (57.78–86.29%), d_10_-Phe (60.61–104.13%), d_12_-Chr (66.91–115.70%), and d_12_-Pyr (73.45–123.88%). After deducting the sample blank, all results were expressed on a dry weight basis.

### 2.4. Positive Matrix Factorization (PMF)

PMF was one of the most wildly applied receptor models of source identification in many studies [17,18,19,20] and the first proposed and applied for source identification by Paatero and Tapper (1994) [21], it was basically established by the following equation:

(1) $$X_{n\times m}=G_{n\times p}\cdot F_{p\times m}+E_{n\times m},$$

where compound concentration matrix $X_{n\times m}$ was decomposed into source contribution matrix $G_{n\times p}$ and source profile matrix $F_{p\times m}$ with residual matrix $E_{n\times m}$. Moreover, *n*, *m*, and *p* represented the number of samples, chemical species, and sources, respectively. The formula can be converted into the following:

(2) $$x_{ij}=\sum_{k=1}^{p}g_{ik}f_{kj}+e_{ij},$$

where $x_{ij}$ was the concentration of the *j*th chemical species detected in the *i*th sample, $g_{ik}$ was the contribution of source *k* to *i*th sample, $f_{kj}$ was the concentration of *j*th chemical species in source *k*, $e_{ij}$ was the residual for each sample and species.

PMF model aimed to find the minimum of the objective function, which was formulated as follows:

(3) $$Q=\sum_{i=1}^{n}\sum_{j=1}^{m}{\left(\frac{e_{ij}}{u_{ij}}\right)}^{2},$$

where $u_{ij}$ was the uncertainty of *j*th chemical specie in the *i*th sample.

The uncertainties for each sample were calculated using measurement uncertainties (MUs) and method detection limits (MDL):

(4) $$u_{ij}=\{\begin{array}{c} \frac{5}{6}\times MDL_{j}\left(x_{ij}\leq MDL_{j}\right)\\ \sqrt{{\left(MU\times x_{ij}\right)}^{2}+{\left(MDL_{j}\right)}^{2}}\left(x_{ij}>MDL_{j}\right) \end{array}.$$

The selection of the *p*-value in the PMF was based on the closeness of a robust Q value (robust Q is the goodness-of-fit parameter calculated excluding outliers) to the theoretical Q value, the diagnostic plots, and relevance of the resolved factors to know physical sources [11]. The theoretical Q value was estimated using the following equation:

(5) $$Q_{\mathrm{theoretical}}=m\times n-p\times \left(m+n\right).$$

In this study, PMF analysis was performed using the U.S. EPA (Environmental Protection Agency) PMF 5.0 model and MU was set to 20%. The dataset used was a 42 × 16 matrix (42 soil samples and 16 individual PAHs), and the model was run in the default robust mode to decrease the influence of extreme values on the PMF solution. *p* was altered between 3 and 10 and eventually set to 4, for which the robust Q values (425.5) best approximate the theoretical Q value (440). The Pearson correlation coefficients (R^2^) between observation and predictand of ∑16PAHs was 0.99, the slope was 1 for linear relation, which indicated the model was well operated.

### 2.5. BaP Toxic Equivalent Concentration

BaP toxic equivalent concentration (BaP_eq_), a common evaluation method, was applied to assess the health risk of PAHs in many studies [10,12,22]. It was calculated by multiplying the concentration of individual PAHs and the corresponding toxicity equivalent factor (TEF) value provided by Nisbet and LaGoy (1992) [23].

(6) $$BaP_{eq}=\sum_{i}^{n}C_{i}\times TEF_{i},$$

where $C_{i}$ was the concentration of *i*th PAH, *n* was the number of individual PAH, $TEF_{i}$ was the corresponding TEF of *i*th PAH.

In order to further interpret the adverse effect of PAHs, population-weighted BaP_eq_ was employed, which was calculated as ratios of population density in each district to average population density multiplied by BaP_eq_ [24]. Population and area were according to Taiyuan statistical yearbook-2017 [25].

### 2.6. Statistical Analysis

Spatial distribution of PAH concentration and risk was mapped out by ArcGIS 10.2 (Esri, Redlands, CA, USA). All statistical procedures including one-way ANOVA and correlation analysis were carried out using SPSS 18 (IBM SPSS, Armonk, NY, USA). Result were considered statistically significant if the *p* value was <0.05.

## 3. Result and Discussion

### 3.1. PAHs Levels

**Figure 1:**
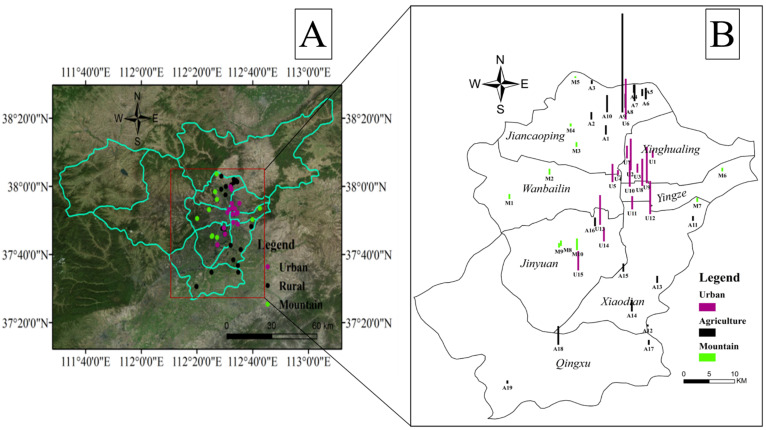
(**A**) The distribution of sampling sites in Taiyuan and (**B**) the level of ∑16PAHs in each site.

The level of U.S. EPA priority individual PAHs, the sum of 16 PAHs (∑16PAHs), and the sum of 7 carcinogenic PAHs (∑7cPAHs) in Taiyuan surface soil samples are listed in Table 1. The concentrations of ∑16PAHs varied from 104.78 to 6594.63 ng g^−1^, with a mean of 922.93 ng g^−1^. The concentrations of ∑7cPAHs ranged from 44.81 to 3534.92 ng g^−1^, with a mean of 478.11 ng g^−1^ accounting for 51.80% of ∑16PAHs. The highest concentrations of the ∑16PAHs (6594.63 ng g^−1^) and ∑7cPAHs (3534.92 ng g^−1^), which was 88 times higher than that of the lowest site (A12: 39.99 ng g^−1^), were detected in soil samples at A9 (Figure 1).

The concentration of ∑16PAHs in the urban soil was the highest among the three types of soil, which ranged from 294.36 to 2540.64 ng g^−1^ with a mean of 1338.60 ng g^−1^. ∑7cPAHs varied from 151.98 to 1285.07 ng g^−1^ accounting for 45.60%–57.72% of ∑16PAHs. On average, the concentration of ∑7cPAHs was 702.12 ng g^−1^, which made up 52.45% of ∑16PAHs. The concentration of ∑16PAHs in urban soil of this study was higher than that of those from Ulsan, South Korea [26], Belgrade, Serbia [27], Viseu, Portugal [28], Shanghai, China [11], Beijing, China [29], Jiangsu Province, China [30], and Yangtze River Delta (YRD), China [31]. However, urban PAH level in this study was lower than those in Lisbon, Portugal [28], Dhanbad, India [32], Nanjing, China [16], and Lanzhou, China[2]. By comparison, it was found that the concentration level of soil PAHs in the Taiyuan urban area was higher than that in coastal or non-heavy industry cities but lower than that in cities with more intensive heavy industry.

**Figure 2:**
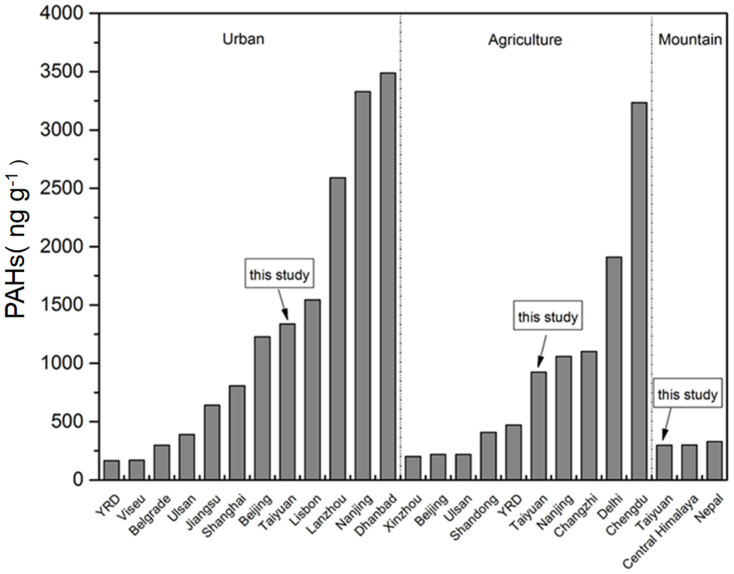
PAH (Polycyclic aromatic hydrocarbon) concentrations (ng g^−^^1^) in urban, agricultural, and montane soil from Taiyuan and other areas.

The concentrations of ∑16PAHs in Taiyuan agricultural soil ranged from 132.82 to 6594.63 ng g^−1^, with a mean of 924.05 ng g^−1^. As shown in Figure 2, ∑16PAHs of agricultural soil in Taiyuan was at moderate level, which was higher than that in YRD [33], Beijing [29], Xinzhou [15], and Shandong Province [34], China and Ulsan, South Korea [26] but lower than that in Nanjing [16], Changzhi [10], and Chengdu [35], China and Delhi, India [3]. The concentration of ∑16PAHs in agricultural soil in Taiyuan was comparable to that in Changzhi but higher than that in Xinzhou although the three cities all belonged to Shanxi province. The industrial structures of these cities were similar, but the enterprise scale of Taiyuan and Changzhi was comparable and larger than Xinzhou, especially in heavy industry. Moreover, in Taiyuan agricultural soil, ∑7cPAHs varied from 39.99 to 3534.92 ng g^−1^ (mean of 476.69 ng g^−1^), which accounted for 30.11%–55.15% (mean of 51.59%) of ∑16PAHs.

Compared with urban and agricultural soil, the lowest concentration of ∑16PAHs was observed in montane soil, which was only 104.78–695.26 ng g^−1^ (on average 297.30 ng g^−1^). Furthermore, concentrations of ∑7cPAHs in montane soil, the lowest values among three types of soil, were 44.81–355.62 ng g^−1^ (on average 144.81 ng g^−1^), accounting for 42.48%–51.92% (on average 48.70%). There was no statistical significance among the three different soils except for that between mountain and urban area (Figure S1). The mountain in this study was close to the city with low altitude, however, the concentration of ∑16PAHs was similar to that of a remote mountain with high altitudes (Figure 2), such as those in Nepal and Central Himalaya [36]. Accordingly, it was reasonable to regard montane soil in this study as the background soil.

The most widely used contamination classification method proposed by Maliszewska-Kordybach (1996) [37] divided soil contaminated by PAHs into four categories: non-contaminated (<200 ng g^−1^), weakly contaminated (200–600 ng g^−1^), contaminated (600–1000 ng g^−1^), heavily contaminated (>1000 ng g^−1^). According to the method, only 6.82% of samples in this study were non-contaminated; 45.45%, 18.18%, and 29.55% of samples were classified as weakly contaminated, contaminated, and heavily contaminated, respectively. Specifically, all the urban soil samples were contaminated, in which 60.00% reached heavily contaminated. Furthermore, 5.27% of the agricultural soil samples were non-contaminated; however, 21.05% were heavily contaminated. No samples were heavily contaminated in montane soil, and weakly contaminated samples accounted for 70.00%.

### 3.2. Compositional Profiles of PAHs

**Figure 3:**
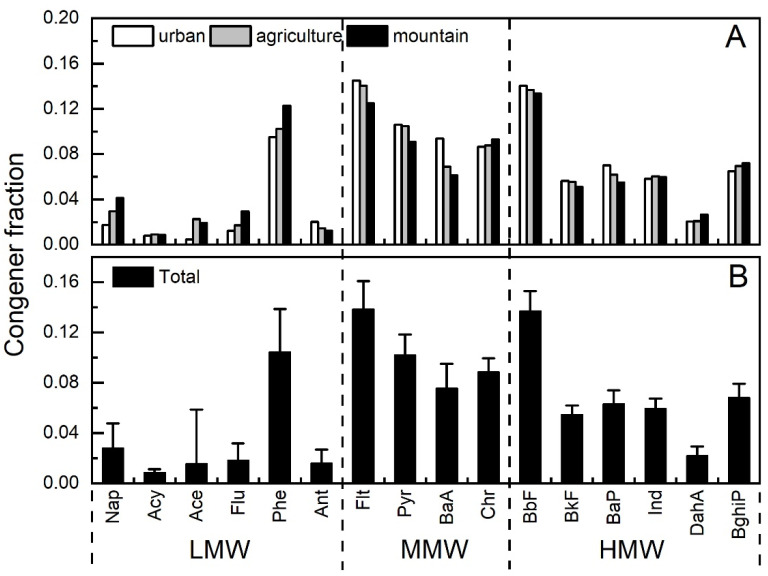
Profile of 16 U.S. EPA (Environmental Protection Agency) PAHs in the soil: (**A**) the mean profile for the urban area, agricultural, and montane soil and (**B**) the mean profile for all the soil samples. The error bars represent standard derivations (SD) across all samples. LMW: low molecular weight, MMW: medium molecular weight, HMW: high molecular weight.

The mean profiles of 16 PAHs in Taiyuan soil are shown in Figure 3. According to molecular weight (MW) of the 16 PAHs, they were divided into three groups: Low MW (LMW)—Nap, Acy, Ace, Flu, Phe, and Ant, Medium MW (MMW)—Flt, Pyr, BaA, and Chr and High MW (HMW) PAHs—BbF, BkF, BaP, Ind, DahA, and BghiP. On the whole (Figure 3B), it can be clearly seen that MMW- and HMW-PAHs were the major components, accounting for 40.45% and 40.47%, followed by LMW-PAHs (19.08%). The proportions of Flt, BbF, Phe, Pyr, Chr, BaA, BghiP, and BaP were higher than the average level of 6.25%, accounting for 13.84%, 13.71%, 10.44%, 10.19%, 8.85%, 7.56%, 6.84%, and 6.31%, respectively. Especially, Flt and BbF were significantly higher than the others (*p* < 0.05) (Figure S2). As shown in Figure 3A, similar PAHs compositional profiles were observed in urban, agricultural, and montane soil. However, higher fractions of LMW-PAHs (23.31%), such as Nap, Ace, Flu, and Phe, and lower fractions of MMW-PAHs (39.74%), such as Flt, Pyr, BaA, and BaP, were observed in montane soil. Moreover, significant differences (*p* < 0.05) between montane and urban area were revealed in terms of Nap, Flu, Flt, Pyr, BaA, and BaP (Figure S3).

For most of the soil samples, the concentrations of MMW and HMW PAHs were higher than those of LMW-PAHs. Furthermore, the fraction of LMW-PAHs increased slightly along the urban (15.77%), agricultural (19.46%), and montane (23.31%) soils. A similar gradient was reported by Wang et al. (2011) [38] in PAH levels in Dalian soils, which could be explained by the “urban fractionation” effect.

### 3.3. Spatial Distribution of PAHs

**Figure 4:**
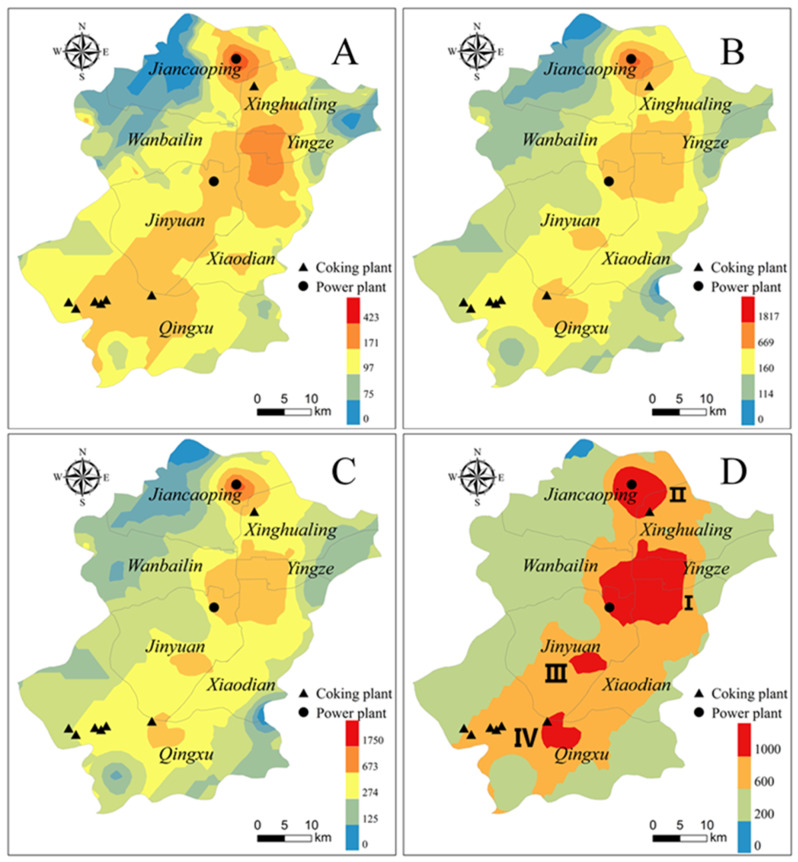
Distribution of LMW-PAHs (**A**), MMW-PAHs (**B**), HMW-PAHs (**C**), and ∑16PAHs (**D**). LMW: low molecular weight, MMW: medium molecular weight, HMW: high molecular weight.

The mean concentration of ∑16PAHs in all soil samples was 922.93 ng g^−1^ with a standard deviation of 1073.76 ng g^−1^, indicating that there was an obvious spatial difference in PAH distribution. In this study, kriging interpolation was applied to map out the spatial distribution characteristics of PAHs in soil with LMW-, MMW-, HMW-PAHs, and ∑16PAHs concentrations as measured values. The simulation results are shown in Figure 1B and Figure 4, respectively. In the spatial distribution of soil PAHs, most of the hotspots were found in the vicinity of factories and urban roads. In the above analysis, the ∑16PAHs concentration of urban soil (1338.60 ng g^−1^) was higher than that of agricultural (924.05 ng g^−1^) and montane (297.30 ng g^−1^) soil. Previous studies on soil pollutants have reported that the concentrations of ∑16PAHs decreased along the urban–suburban–rural gradient in several areas, such as Beijing [29], Nanjing [16], Pearl River Delta (PRD) [39], Ulsan, South Korea [26], which was caused by variations of urbanization and economic development level [29].

Urban areas were hot spots for PAH pollution, and concentrations of ∑16PAHs higher than 1000 ng g^−1^ were measured in 9 samples of all the 15 urban soil samples, with concentrations of U6, U12, and U13 exceeding 2000 ng g^−1^ (Figure 1). The reason was that many industries were located around the urban area, including thermal power plants, steel plants, and coking plants (Figure 4), and PAHs emitted from these plants could transport and diffuse into urban areas. The highest level was detected in U6 (2540.64 ng g^−1^) collected from a park in the vicinity of a thermal power plant and a coking plant. U12, the second-highest site with ∑16PAHs concentration of 2271.56 ng g^−1^, was from a park in Xiaodian district (Figure 1). The park was located next to the main traffic trunk road with heavy traffic and traffic jams, which caused large amounts of PAHs [29]. The long-term high exhaust emissions from vehicle, deposition, and accumulation into soil led to higher PAH concentrations in U9 [29]. The concentration of ∑16PAHs of U13 was 2067.01 ng g^−1^ collected near a chemical industrial park and a thermal power plant. The lowest concentration of PAHs was observed in U4 (294.36 ng g^−1^) collected from a new park, which is far from factories and the trunk road. Moreover, the low level of soil PAHs in new parks was thought to be associated with covered soils transported from other clean areas [40].

For agricultural soil, A9 (Figure 1) was the highest site in all samples with ∑16PAHs concentration of 6594.63 ng g^−1^ and was also the closest site to the thermal power plant and coking plant where the U6-site is located (Figure 4). On the other hand, a large number of diesel trucks passed by the agricultural area, carrying coal required for industrial production. In addition to the sample with the highest concentration, the average ∑16PAHs level of agricultural soil in northern Taiyuan (9 samples, 246.55–1114.95 ng g^−1^, mean of 705.05 ng g^−1^) was higher than that in southern Taiyuan (9 samples, 132.82–1248.65 ng g^−1^, mean of 512.98 ng g^−1^), indicating that the soil pollution in northern Taiyuan was slightly higher than that in the southern part. As for all agricultural soil samples in the whole city, higher concentrations of ∑16PAHs were obtained from these sites surrounding plants, including a thermal power plant and two coking plants (Figure 4), which suggests that these areas were greatly influenced by the industry source.

In montane soil, the highest concentration of ∑16PAHs was found in M10 (695.26 ng g^−1^), the nearest sampling point to the urban area (Figure 1), indicating that the site may be vulnerable to urban emission diffusion [29]. The ∑16PAHs concentration in the montane soil was relatively low after exclusion of M10-site (104.78–395.26 ng g^−1^, mean of 253.08 ng g^−1^), and the SD was small (SD = 84.57), which means that the PAHs in montane soils were at a low level, and the spatial distribution difference was small.

Four heavily contaminated zones (HCZs) (I–IV) were presented in the spatial distribution of ∑16PAHs (Figure 4D) according to the contamination classification method proposed by Maliszewska-Kordybach (1996) [37]. HCZ I and HCZ III were located in downtown and HCZ II and IV were near two industrial parks (Figure 4D). The spatial distribution regions of MMW- (Figure 4B) and HMW-PAHs (Figure 4C) were the same and similar to the distribution of ∑16PAHs, but some different characteristics were revealed in the spatial distribution of LMW-PAHs (Figure 4). In general, LMW-PAHs mainly come from petrogenic sources, while MMW- and HMW-PAHs mainly come from pyrolytic sources [41]. The high emissions of pyrolytic sources were the dominant factor of the four HCZs due to higher-ring PAHs (≥4 rings), which are prone to accumulate in soils close to emission sources [2]. In the spatial distribution of LMW-PAHs (Figure 4A), not only HCZ III and IV disappeared, but also the concentration of ∑16PAHs in HCZ I and II was lower. This phenomenon may be caused by the low emission and physicochemical properties of LMW-PAHs. LMW-PAHs are more volatile and susceptible to long-distance transport in the gas phase [5,12]. Even accumulated in the soil, LMW-PAHs may re-volatilized from the surface soil and return to the atmosphere in warm seasons [42]. In addition, LMW-PAHs may be more sensitive to photochemical degradation, microbial degradation, and plant absorption in environmental media.

TOC was generally used as an important factor influencing the spatial distribution pattern of PAHs in soil, which dominated the sorption and the fate of organic compounds [2,3,12]. In this study, the mean TOC in soil was 4.52% and ranged from 2.70% to 9.63%. A Pearson correlation coefficient was employed to evaluate the relationship between TOC and PAH concentrations. There was no significant correlation between TOC and concentrations of ∑16PAHs (*p* > 0.05), which may be because adsorption equilibrium between TOC and PAHs was not reached with continuous input of fresh PAHs [11,12]. Furthermore, there existed significant correlation (r = 0.332, *p* = 0.028) between TOC and LMW-PAHs rather than among TOC and MMW- (r = 0.274, *p* > 0.05) and HMW-PAHs (r = 0.273, *p* > 0.05). Generally speaking, the influence of TOC on soil PAH spatial distribution was ignored.

### 3.4. Source Identification by PMF

**Figure 5:**
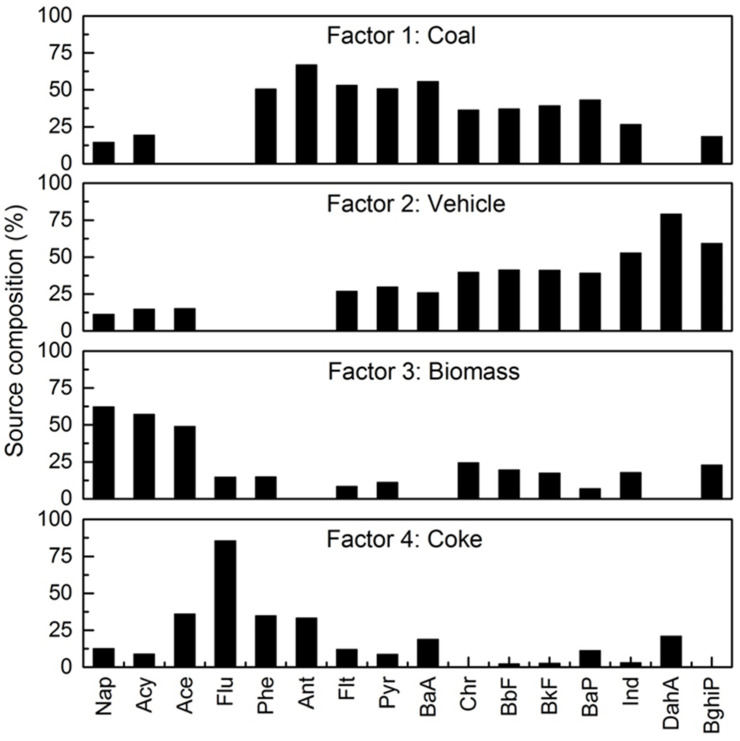
Source profiles obtained by PMF (positive matrix factorization) model.

In order to investigate the source and contribution of PAHs in soils, a PMF model was employed to identify the sources. As shown in Figure 5, four factors were obtained by the PMF model with their source profiles. Factor 1 was dominated by Phe, Ant, Flt, Pyr, BaA, and Bap, which were all the indicators of coal combustion [17,18,19,20]. Therefore, factor 1 identified the source as coal combustion. Factor 2 was mainly loaded on Ind, DahA, and BghiP. Ind and BghiP are reported as typical tracers of vehicle sources [18,20], and DahA is a typical compound for diesel emissions [19]. Thus, factor 2 could be interpreted as a vehicle source. Nap, Acy, and Ace dominate in factor 3. Acy is the predominant individual PAH emitted from the biomass combustion process [43,44], and Nap is generally attributed to the incomplete combustion process of the combustion-related source [19]. So, factor 3 identified the source as biomass combustion. Factor 4 was heavily weighted on Flu, which was recognized as a tracer produced by coking furnace [19,20]. Therefore, factor 4 represented the coking source.

**Figure 6:**
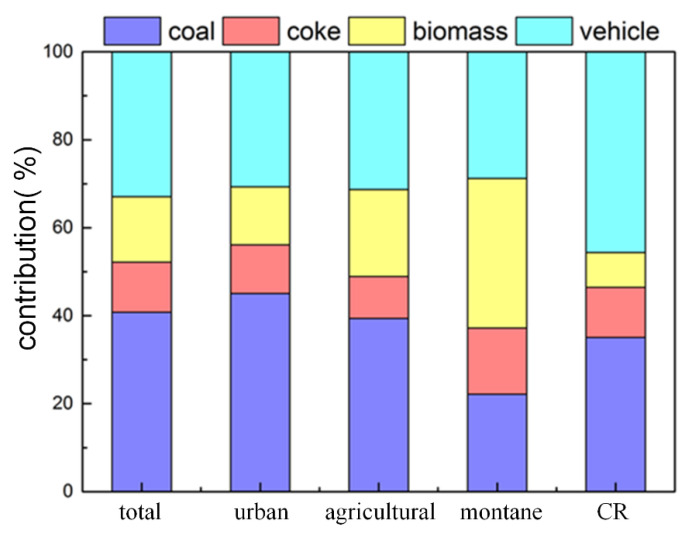
Contribution profile of total, urban, agricultural, montane samples, and carcinogenic risk (CR).

The contribution profiles of the PAHs in total, urban, agricultural, and montane soils are shown in Figure 6. In total soils, the relative contributions of the four identified sources to the total PAHs followed the order of coal combustion (40.77%), vehicle (32.94%), biomass burning (14.89%), and coke (11.40%) (Figure 6). It is evident that coal-related sources (coal and coke: 52.17%) were the major source of PAHs in soils from Taiyuan. Coal, the primary energy in Taiyuan, was widely used in the daily life of residents and in industrial activities, such as coking, coal-fired heating, which was the main contributor to the PAH emission inventory of Taiyuan [45]. Meanwhile, the coal-related source was reported as the major source in atmospheric PAHs of Taiyuan, such as gas phase and total suspended particulate [5], PM_2.5_ [6], dry deposition [7], and rainwater [46]. Therefore, policy-makers should give priority to adjust energy structure to reduce soil environmental pollution, which was the leading factor to improve PAH pollution in Taiyuan.

In urban and agricultural soils (Figure 6), coal source (45.05%, 39.39%) and vehicle sources (30.70%, 31.33%) were dominant contributors among the four sources, followed by biomass sources (13.20%, 19.76%) and coking sources (11.05%, 9.52%). Although the order of source contributions was the same, the contributions of coal and biomass sources varied oppositely, and other sources hardly changed. In some rural areas, more coal replaced by biomass (straw and fuelwood, etc.) as fuel for winter heating and cooking, and agricultural incineration was also common. Under the influence of these factors, higher biomass source contributions were observed in some agricultural soils. For example, the biomass source contributions of A12, A3, A11, and A17 were 34.10%, 33.72%, 33.23%, and 32.01%, respectively. These agricultural areas were far from urban and industrial areas, and greatly affected by local sources.

PAHs in montane soil mainly came from the transmission and diffusion of urban and rural emissions. The source contribution of montane soils showed quite different results from those of urban and agricultural areas: biomass source (34.05%) > vehicle source (28.76%) > coal source (22.15%) > coking source (15.05%) (Figure 6). Agricultural areas are generally closer to the mountain than urban areas, which contributed more PAHs to montane areas because biomass combustion was mainly concentrated in rural areas. Montane soil samples with high biomass source contributions such as M15 (43.22%) and M7 (38.29%) were close to the agricultural soils A3 and A11, respectively. In addition, LMW-PAHs, with volatility and capacity for long-distance transmission, were mainly emitted from biomass combustion in the rural area and then sunk into the montane soil owing to the influence of the basin topography [47]. This led to the different distribution of montane soil source profiles compared with urban and agricultural areas.

Generally speaking, coal-related sources were the main sources of PAHs in urban areas (56.10%), agricultural (48.91%), and montane soils (37.20%). The proportion was higher especially in soils with severe PAH pollution, such as the sample with the higher Σ16PAHs concentration in urban (U6, 52.22%), agricultural (A9, 62.11%), and montane (M10, 43.64%) soils.

### 3.5. Human Health Risk Assessments of PAHs in Soils

BaP_eq_ was applied to assess the health risk of PAHs in soil. The BaP_eq_ concentration of the ∑16PAHs in urban soil samples was 41.29–295.90 ng g^−1^, with an average value of 169.59 ng g^−1^ (Table 2). In agricultural soil samples, the BaP_eq_ concentration ranged from 8.23 to 955.05 ng g^−1^, with a mean of 119.48 ng g^−1^. BaP_eq_ concentrations ranged from 13.32 to 79.74 ng g^−1^ (mean value was 33.63 ng g^−1^) in montane soils. BaP_eq_ in urban area was significantly higher than that of mountain (*p* < 0.05), while there was no significant difference between agricultural soils and urban or montane soil (Figure S4).

**Figure 7:**
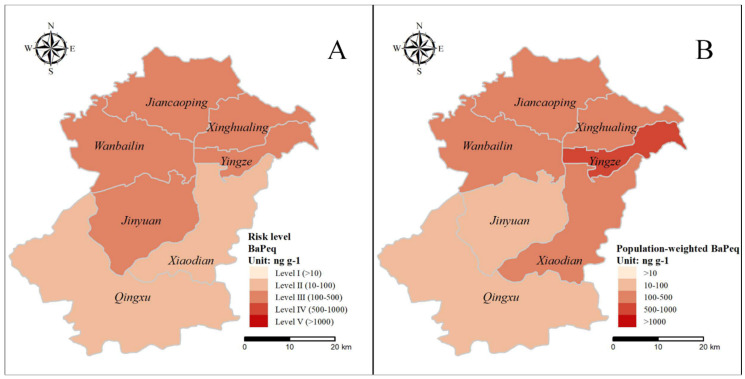
(**A**) Spatial distributions of risk level and (**B**) population-weighted BaP_eq_ in soils.

According to the soil quality standards applied by Li et al. (2016) [22], the BaP toxicity equivalent concentration of ∑16PAHs was divided into five levels of carcinogenic risk: (I) BaP_eq_ ≤ 10 ng g^−1^ (no risk); (II) 10 ng g^−1^ < BaP_eq_ ≤ 100 ng g^−1^ (low risk); (III) 100 ng g^−1^ < BaP_eq_ ≤ 500 ng g^−1^ (medium risk); (IV) 500 ng g^−1^ < BaP_eq_ ≤ 1000 ng g^−1^ (high risk); and (V) BaP_eq_ > 1000 ng g^−1^ (extremely high risk). Ten urban samples (66.67%) were at medium risk, and the other five samples (33.33%) were at low risk, which means that there was a carcinogenic risk in the soil of urban parks in Taiyuan, and most regions were threatened by medium carcinogenic risk. The number of agricultural soil samples with no risk, low risk, medium risk, and high risk were 1 (5.26%), 13 (68.42%), 4 (21.06%), and 1 (5.26%), respectively. Agricultural soils with medium and high risk were A7, A8, A10, A18, and A9. These soils were located in the vicinity of power plants and coking plants. Therefore, industrial activities pose a certain carcinogenic threat to agricultural soil and food safety, thereby posing threats to human health in Taiyuan. All montane samples were at low risk of carcinogenesis. To better understand the carcinogenic risk. To better understand the carcinogenic risk to population, population density was introduced to address the overall risk by calculating the population-weighted BaP_eq_. As previously mentioned, montane soils were regarded as the background of urban soil with significantly lower BaP_eq_ than urban soil, moreover, the population density is generally lower than that in downtown and countryside. In further discussion, BaP_eq_ in montane soils was ignored. The district-based BaP_eq_ was calculated, then multiplied corresponding population-weight ratio. The results found that BaP_eq_ in Yingze and Xiaodian were elevated and the BaP_eq_ in Jinyuan was moderate when population density was considered (Figure 7B).

In order to determine the major contributors to the carcinogenic risk of soil PAHs, the results of PMF model was combined with BaP_eq_. As shown in Figure 6, coal usage (46.48%) was the main source of contribution for BaP_eq_, followed by vehicle exhausts (45.64%), and the lowest contribution was biomass (7.88%). The spatial distribution of carcinogenic risk levels (Figure 7A) was comparable to that of ∑16PAHs (Figure 4), indicating that carcinogenic risk was related to the concentration of ∑16PAHs. Moreover, different emission sources lead to a difference of carcinogenic risk levels. PAHs emitted by coal combustion and vehicle exhausts were more carcinogenic because these sources were characterized by PAHs with a high value of TEF [18,20], leading to higher BaP_eq_ concentrations and higher levels of carcinogenic risk [23]. Combined with the spatial distribution of risk level, both urban and industrial areas dominated by these two sources were mainly at level III risk (Figure 7A). As shown in Figure 7B, the risks in there were emphasized due to the dense population. Therefore, people living in there may be affected by more carcinogenic effects of PAHs. Policy-makers need to work out reasonable and practical measures of soil protection to ensure human health.

## 4. Conclusions

The concentrations of∑16PAHs in Taiyuan soil ranged from 104.78 to 6594.63 ng g^−1^ with a mean concentration of 922.93 ng g^−1^. The average concentration of soil ∑16PAHs (1338.60 ng g^−1^) in the urban area was significantly higher than that of its surrounding soil (agriculture: 924.05 ng g^−1^; mountain: 297.30 ng g^−1^). According to the classification of contaminated soils proposed by Maliszewska-Kordybach (1996) [37], 6.82% of all soil samples were uncontaminated, 45.45% were weakly contaminated, 18.18% were contaminated, and 29.55% were heavily contaminated. In all, 60.00% of urban soil and 21.05% of agricultural soil showed heavy pollution levels, which need to be paid serious attention. Higher-molecular-weight PAH homologues (≥4 rings) were dominant in PAHs profiles accounting for 80.92%. Similar compositional profiles were also observed in urban, agricultural, and montane soils. In the spatial distribution of PAHs, urban and industrial areas were the most polluted areas. The PMF model was applied and four major sources were obtained in Taiyuan soil: coal (40.77%), vehicle (32.94%), biomass (14.89%), and coking (11.40%). The risk assessment of soil conducted by BaP_eq_ suggested that the higher carcinogenic risk of soil was found in the urban area and the vicinity of the industry. Coal usage (coal + coking) was the primary factor of carcinogenic risk to the soil in Taiyuan (46.48%), followed by vehicle exhausts (45.64%). It is reasonable to give priority to adjusting energy structure dominated by coal for reducing soil carcinogenic risk, thereby ensuring agricultural-product safety and human health.

## Figures and Tables

**Table 1:** Concentrations of polycyclic aromatic hydrocarbons (PAHs) in different types of surface soils from Taiyuan (ng g^−1^).

Compounds	Urban Soils				Agricultural Soils				Montane Soils			
	Min	Max	Mean	SD	Min	Max	Mean	SD	Min	Max	Mean	SD
Nap	8.35	38.67	19.53	9.53	9.29	31.26	14.65	5.55	6.13	17.97	11.18	3.92
Acy	1.94	23.58	11.00	6.87	1.42	55.89	7.70	11.94	1.08	3.88	2.36	0.90
Ace	1.84	13.24	5.61	3.39	1.48	71.11	7.97	15.73	0.71	15.53	5.20	4.08
Flu	6.26	42.40	14.72	9.21	3.83	30.16	9.36	5.92	3.94	14.48	7.97	3.50
Phe	24.44	324.52	128.31	80.13	18.61	510.73	81.29	107.66	17.17	80.96	35.75	18.62
Ant	3.74	149.62	30.41	36.35	3.65	77.97	12.21	16.86	1.49	8.44	3.53	1.82
Flt	37.85	424.45	200.46	120.64	9.31	1090.25	143.94	236.50	9.24	99.04	38.38	23.46
Pyr	33.15	308.48	144.02	83.56	6.32	816.54	107.35	177.14	6.87	65.52	27.38	15.14
BaA ^c^	24.04	256.24	131.24	78.95	4.19	597.21	73.07	129.90	5.07	45.99	18.72	11.21
Chr ^c^	24.79	209.74	113.31	55.82	8.70	521.56	78.88	111.03	6.65	73.05	28.71	17.52
BbF ^c^	37.64	350.47	185.73	93.52	12.79	896.81	127.58	191.56	11.44	106.22	41.03	25.56
BkF ^c^	16.43	156.38	75.64	40.84	4.00	392.74	53.61	84.34	4.01	36.56	15.39	8.61
BaP ^c^	20.72	158.04	94.03	49.65	3.72	543.43	65.57	118.01	5.49	41.04	16.60	9.64
Ind ^c^	18.06	144.87	76.29	38.45	4.94	414.53	57.12	88.76	7.31	38.31	17.23	8.24
DahA ^c^	9.83	45.64	25.89	12.66	1.66	168.64	20.85	36.55	4.68	14.45	7.14	3.07
BghiP	24.81	148.13	82.42	38.77	6.85	431.22	62.89	91.82	8.28	44.00	20.74	9.65
∑7cPAHs	151.98	1285.07	702.12	362.94	39.99	3534.92	476.69	759.33	44.81	355.62	144.81	82.96
∑16PAHs	294.36	2540.64	1338.60	709.18	132.82	6594.63	924.05	1410.44	104.78	695.26	297.30	157.11

^c^ Carcinogenic PAHs.

**Table 2:** BaP toxic equivalent concentration (BaP_eq_) concentration of 16 PAHs in soils from Taiyuan.

PAHs	TEFs	Urban	Agricultural	Montane
Nap	0.001	0.02	0.01	0.01
Acy	0.001	0.01	0.01	0.00
Ace	0.001	0.01	0.01	0.01
Flu	0.001	0.01	0.01	0.01
Phe	0.001	0.13	0.08	0.04
Ant	0.01	0.30	0.12	0.04
Flt	0.001	0.20	0.14	0.04
Pyr	0.001	0.14	0.11	0.03
BaA	0.1	13.12	7.31	1.87
Chr	0.01	1.13	0.79	0.29
BbF	0.1	18.57	12.76	4.10
BkF	0.1	7.56	5.36	1.54
BaP	1	94.03	65.57	16.60
Ind	0.1	7.63	5.71	1.72
DahA	1	25.89	20.85	7.14
BghiP	0.01	0.82	0.63	0.21
Total	-	169.59	119.48	33.63

TEFs: toxicity equivalent factors.

## Supplementary Materials

The following are available online at https://www.mdpi.com/1660-4601/17/17/6319/s1, Figure S1: The concentration for the urban area (U), agriculture (A), and mountain (M). Figure S2: The concentration of individual PAH in this study. Figure S3: The concentration of individual PAH in the urban area (U), agriculture (A), and mountain (M). Figure S4: The BaP_eq_ in the urban area (U), agriculture (A), and mountain (M).
