# Supplementary material for: Distribution of the Soil PAHs and Health Risk Influenced by Coal Usage Processes in Taiyuan City, Northern China
Source: Int J Environ Res Public Health. 2020 Aug 31;17(17):6319. doi: 10.3390/ijerph17176319 (PMC7503818; doi:10.3390/ijerph17176319)
Supplement: Supplementary file 1 [file ijerph-17-06319-s001.pdf]

Supporting Material of

Distribution of the Soil PAHs and Health Risk Influenced by Coal Usage Processes in Taiyuan City, Northern China

Rongjie Li [1], Mingchao Cheng [1], Yang Cui [1], Qiusheng He [1,*], Xiaofang Guo [1], Ming Liu [2], Laiguo Chen [2,*] and Xinming Wang [3]

[1] School of Environment and Safety, Taiyuan University of Science and Technology, Taiyuan 030024, China; tyustlrj@163.com (R.L.); cheng.mingchao@foxmail.com (M.C.); cuiyang@mail.iap.ac.cn (Y.C.); guoxiaofang@tyust.edu.cn (X.G.)
[2] Center of Urban Air Pollution, South China Institute of Environmental Science (SCIES), Ministry of Ecology and Environment of the People's Republic of China, Guangzhou 510655, China; chenlaiguo@scies.org
[3] State Key Laboratory of Organic Geochemistry, Guangzhou Institute of Geochemistry, Chinese Academy of Sciences, Guangzhou 510640, China; wangxm@gig.ac.cn
* Correspondence: heqs@tyust.edu.cn; Tel.: +86-351-6998326; Fax: +86-351-6998326

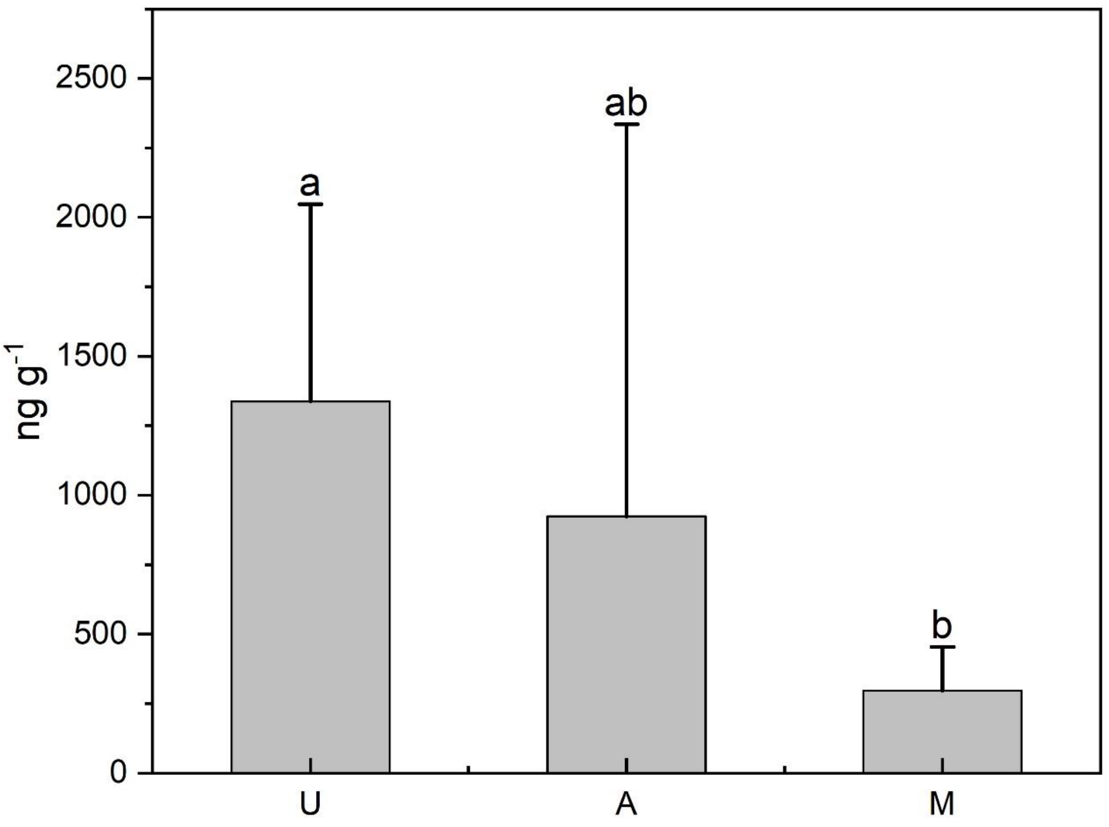

Figure S1. The concentration for the urban area (U), agriculture (A) and mountain (M). The error bars represent standard derivations (SD) across all samples.

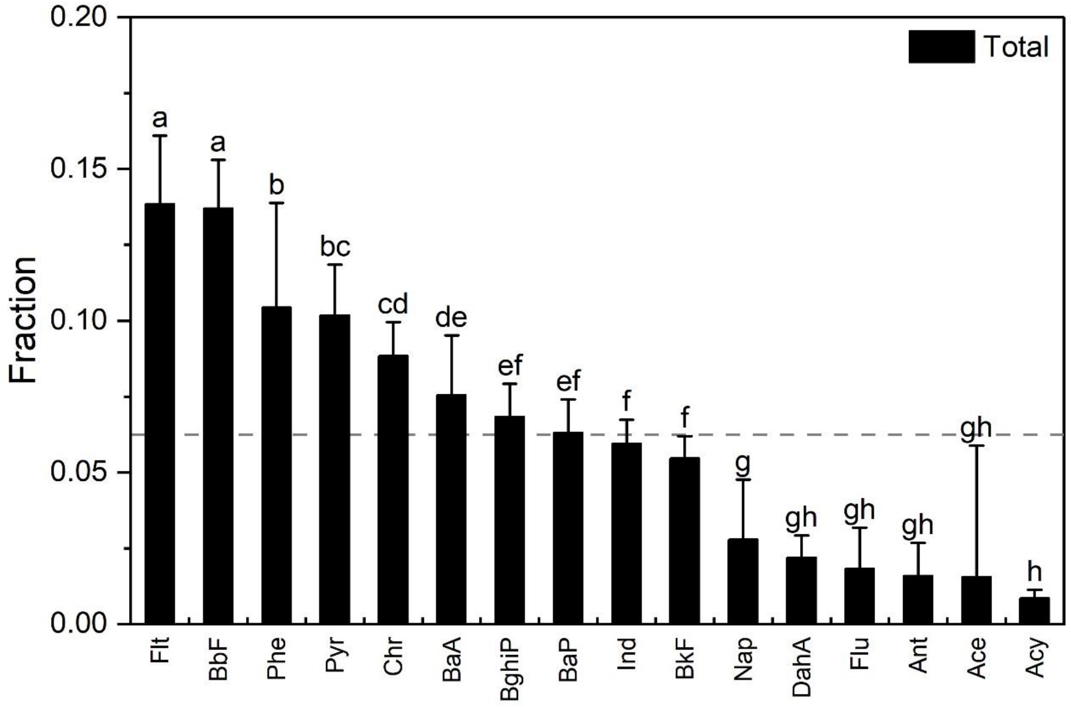

Figure S2. The concentration of individual PAH in this study. The error bars represent standard derivations (SD).

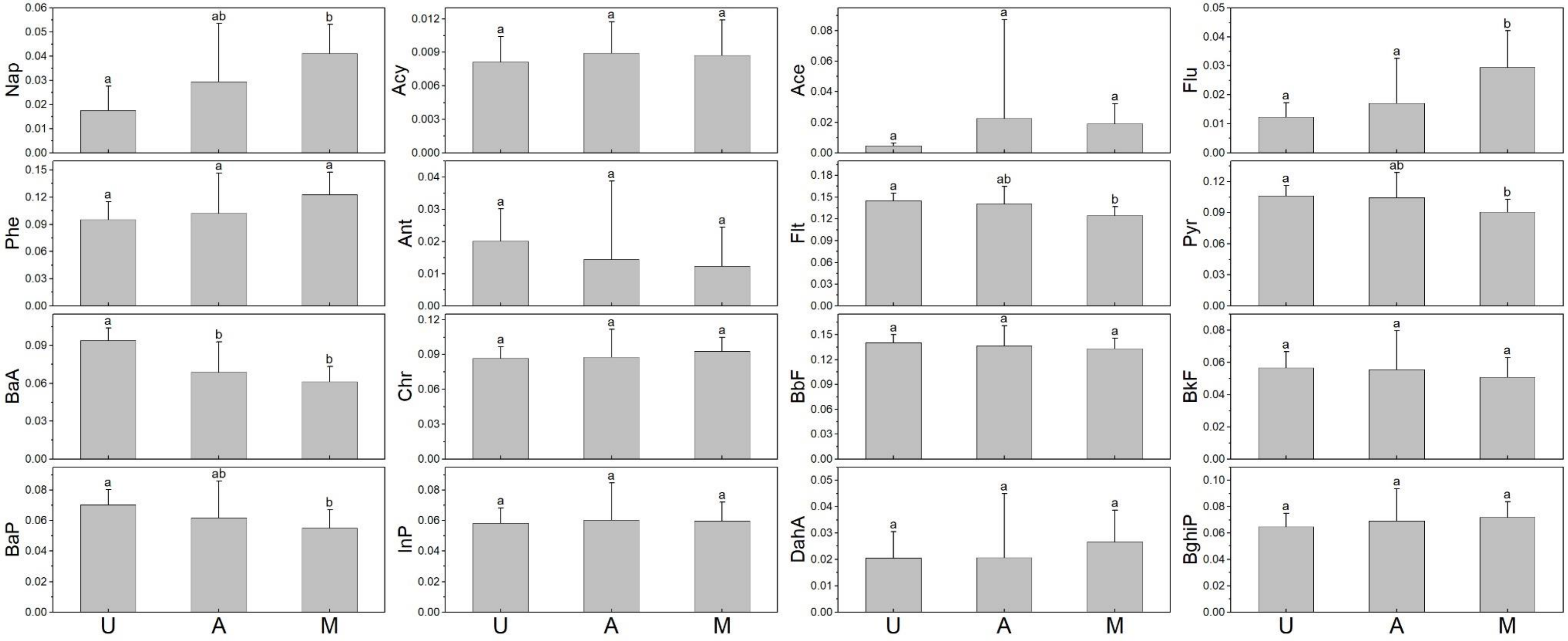

Figure S3. The concentration of individual PAH in the urban area (U), agriculture (A) and mountain (M). The error bars represent standard derivations (SD).

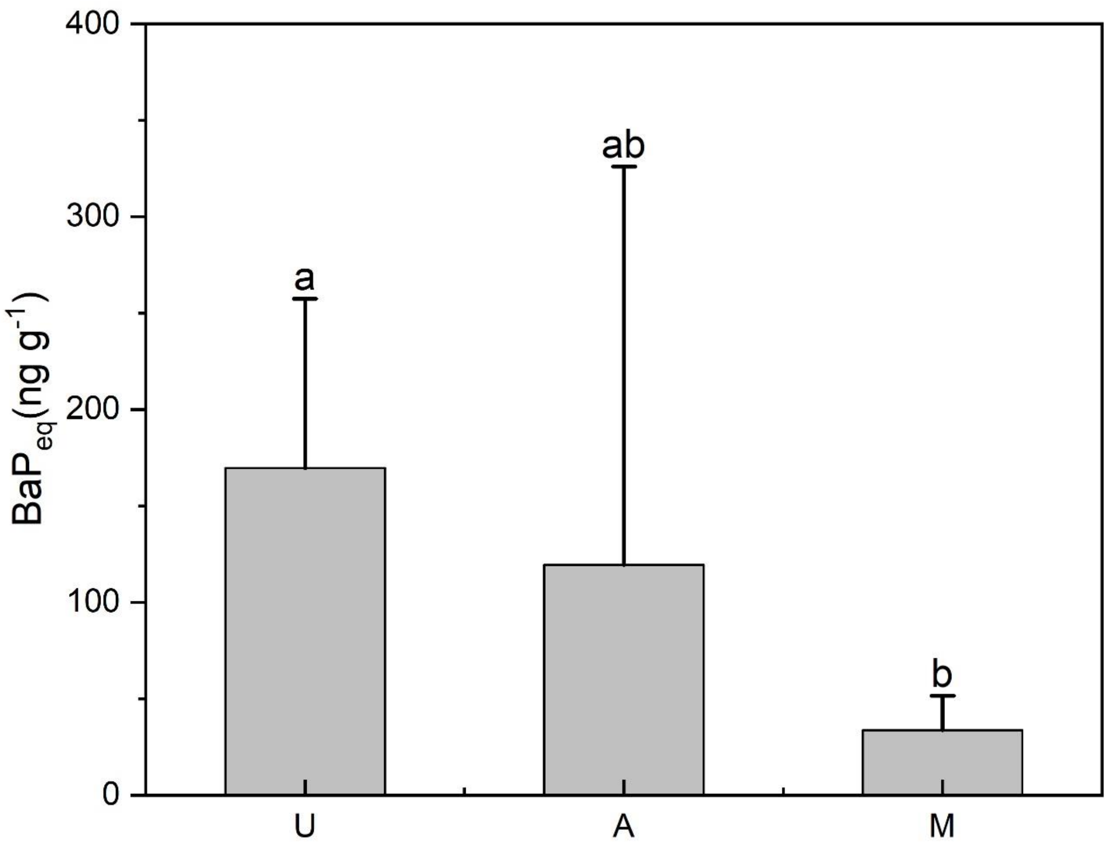

Figure S4. The BaP_{eq} in the urban area (U), agriculture (A) and mountain (M). The error bars represent standard derivations (SD).
